# Immunohistochemical characterization of subtypes of male breast carcinoma

**DOI:** 10.1186/bcr2258

**Published:** 2009-05-14

**Authors:** Yimin Ge, Nour Sneige, Mahmoud A Eltorky, Zhiqin Wang, E Lin, Yun Gong, Ming Guo

**Affiliations:** 1 Department of Pathology, The Methodist Hospital, Weill Medical College of Cornell University, 6565 Fannin Street, Houston, Texas 77030, USA; 2 Department of Pathology, The University of Texas M. D. Anderson Cancer Center, 1515 Holcombe Boulevard, Houston, Texas 77030, USA; 3 Department of Pathology, University of Texas Medical Branch, 301 University Boulevard, Galveston, Texas 77555, USA; 4 Department of Biostatistics and Applied Mathematics, The University of Texas M. D. Anderson Cancer Center, 1515 Holcombe Boulevard, Houston, Texas 77030, USA

## Abstract

**Introduction:**

Male breast cancer accounts for around 1% of all breast cancer cases but the incidence has risen in recent years. This study aimed to classify the molecular subtypes of male breast cancers based on the expression profile of immunomarkers and to evaluate their association with clinicopathological features and expression patterns of epidermal growth factor receptor (EGFR) and nuclear factor κB (NF-κB).

**Methods:**

A total of 42 cases of male breast carcinoma were examined retrospectively using immunostains for estrogen receptor (ER), progesterone receptor (PR), cytokeratin 5/6 (CK5/6), EGFR, and NF-κB. Human epidermal growth factor receptor 2 (HER2) expression was evaluated by immunostaining and confirmed by fluorescent *in situ *hybridization (FISH).

**Results:**

The luminal A subtype was the most common subtype in male breast cancer (83%, 35/42), which was followed by the luminal B subtype (17%, 7/42). Basal-like and HER2+/ER- subtypes were not identified in this group. All carcinomas expressed ER and 67% of them were PR+. High nuclear grades were more common in the luminal B subtype (71%, 5/7) than in the luminal A subtype (34%, 12/35). The luminal B subtype carcinomas expressed EGFR (42%, 3/7) and NF-κB (57%, 4/7) more frequently than the luminal A subtype did (17%, 6/35 and 37%, 13/35, respectively).

**Conclusions:**

In our study group, luminal A and B subtypes were the major subtypes of male breast carcinoma. The immunophenotypical features of male breast cancer differ from those of its female counterpart. Luminal B subtype tended to have high nuclear grade and more frequent expression of EGFR and NF-κB.

## Introduction

Male breast cancer is an uncommon disease, accounting for approximately 1% of all breast cancer cases and less than 1% of all malignancies in men [1,2]. The American Cancer Society estimates that 1,910 men will be diagnosed with breast cancer in the USA in 2009 and about 440 men will die from the disease [3]. Although breast carcinoma in both genders shares certain characteristics, there are notable differences in incidence, age distribution, prognosis and survival. The unfavorable overall prognosis in male breast cancer has been attributed to the older age and advanced tumor stage at the time of diagnosis [4].

Based on the recent DNA microarray studies on female breast cancer cases, distinct molecular subtypes of breast carcinoma were identified with different clinical outcomes [5]. Using an intrinsic set of 534 genes, Sorlie and colleagues [6] analyzed the expression profiles of 115 independent breast cancer specimens and categorized them into the following subtypes: luminal; human epidermal growth factor receptor 2 (HER2) over-expressing; normal breast-like; and basal-like. The tumor subtypes correlated well with clinical outcomes as measured by overall survival and distal metastasis, with the worst outcome observed in HER2 over-expressing and basal-like subtypes [5-7].

The molecular subtypes of female breast cancer were originally identified by gene expression analysis using DNA microarrays. However, large-scale subtyping using gene expression profiling from formalin-fixed, paraffin-embedded samples is not currently feasible. Therefore, immunohistochemical (IHC) markers have been used as surrogates for DNA microarray in subtyping breast cancer. By using a panel of four antibodies including estrogen receptor (ER), HER1, HER2 and cytokeratin 5/6 (CK5/6), Nielsen and colleagues [8] characterized three IHC-defined subtypes: luminal (ER+, HER2-), HER2 (HER2+), and basal-like (ER-, HER2-, CK5/6+ or HER1+). Based on more recent gene expression studies, Carey and colleagues [9] updated IHC subtype definition as luminal A (ER+ and/or progesterone receptor (PR)+, HER2-), luminal B (ER+ and/or PR+, HER2+), HER2+/ER- (ER-, PR-, HER2+), basal-like (ER-, PR-, HER2-, CK5/6+), and unclassified (negative for all five markers).

In addition to these subtypes, the prognosis of breast cancer may be affected by expressions of two additional molecular markers. Epidermal growth factor receptor (EGFR) was reported to be over-expressed in aggressive female breast cancer [10-13] and is currently being evaluated in clinical trials as a potential therapeutic target [14-16]. It was also reported that transcription factor nuclear factor κB (NF-κB) was activated in breast cancer cells with over-expressed EGFR [17]. The elevation of NF-κB was speculated to be associated with unfavorable prognosis by promoting metastasis [18,19], inhibiting apoptosis [20], and increasingly resistance to chemotherapy [21]. Increasing evidence has suggested that NF-κB is a potential target for the treatment of breast cancer and the prevention of metastasis [22,23].

Despite these advances in female breast carcinoma, our understanding and treatment strategies for male breast cancer are limited and generally extrapolated from our knowledge of female breast cancer. In particular, the molecular subtypes of male breast cancer have not been studied although the subtypes were reportedly associated with both biological and clinical behaviors in female breast cancer. This article reports our attempt to subclassify male breast carcinomas based on the immunoprofile, and to evaluate the association of the subtypes with histologic type, nuclear grade, lymph node status, clinical stage, and expression of EGFR and NF-κB.

## Materials and methods

### Specimens

Slides and paraffin-embedded tissue blocks from 42 male patients with breast cancer were retrieved from the surgical pathology archives in the departments of pathology of The University of Texas M. D. Anderson Cancer Center (from 2002 to 2005, Houston, Texas) and the University of Texas Medical Branch (from 1990 to 2004, Galveston, Texas). Clinical data for these patients were collected with approval of the Institutional Review Board (IRB# LAB05-0153).

### Histologic evaluation

The slides from these cases were reviewed and the histologic diagnoses recorded in the medical record were confirmed independently by two pathologists (YMG, MG). The histologic classification was based on the criteria set by the World Health Organization. A diagnosis of invasive papillary carcinoma is rendered when a frankly invasive carcinoma predominantly in a papillary growth pattern, or in a ductal/solid patterns but associated with an *in situ *component of papillary carcinoma. The modified Black's nuclear grading system was used for nuclear grading with a three-tire scheme based on abnormalities in nuclear size, nuclear membrane, chromatin, and mitosis.

### Immunohistochemistry and interpretation

Tissue sections (4 μm) from each case were prepared for immunostaining.

After incubation in a 60°C oven overnight and deparaffinization, the tissue sections were treated with 3% hydrogen peroxide in methanol for five minutes. Following a brief pretreatment with 0.02% protease XXIV for two minutes, the slides were incubated with one of the following antibodies: anti-human ER, PR, HER2, CK5/6, EGFR, and NF-κB. The sources and characterization of these antibodies are detailed in Table 1. The immunostains were performed using an automated staining system (BenchMark XT, Ventana Medical System Inc., Tucson, AZ, USA). Mouse Envision (DAKO Corp., St. Barbara, CA, USA) was used as secondary antibody. The color was visualized by incubation with chromagen DAB for 16 minutes. The slides were then counterstained with Mayer hematoxylin and a coverslip with Permount (Fisher Scientific, Fair Lawn, NJ, USA) was placed on the slide.

**Table 1:** Antibody characterization, dilutions and positive controls

**Antigen**	**Vendors**	**Species (clone)**	**Dilution**	**Positive control**
ER	Novocastra Lab Ltd. Newcastle, UK	Mouse IgG1 (6F11)	1:50	Breast
PR	NeoMarkers Inc. Fremont, CA, USA	Mouse IgG1 (1A6)	1:30	Breast
HER2	NeoMarkers Inc. Fremont, CA, USA	Mouse IgG1 (e2-4001)	1:100	Breast carcinoma
CK 5/6	Deko Inc, Carpinteria, CA, USA	Mouse IgG1 kappa (D5/16 B4)	1:100	Skin
EGFR	Zymed Lab Inc. San Francisco, CA, USA	Mouse IgG1 (31G7)	1:50	Squamous cell carcinoma
NF-κB p56	Abcam Inc. Cambridge, MA, USA	Rabbit polyclonal IgG	1:80	Large B cell lymphoma

CK = cytokeratin; EGFR = epidermal growth factor receptor; ER = estrogen receptor; HER2 = human epidermal growth factor receptor 2; NF-κB = nuclear factor-κB; PR = progesterone receptor.

The staining patterns and intensities for each of the markers were interpreted by two pathologists independently (YMG, MG). For ER and PR, nuclear staining in more than 10% of tumor cells was classified as positive staining. A positive HER2 stain was determined by greater than 2+ membranous staining of tumor cells based on the conventional three-tier grading criteria. All cases with greater than 2+ HER2 immunostain were further confirmed by fluorescence *in situ *hybridization (FISH) study. An estimation of more than 10% of tumor cells with membranous or cytoplasmic staining was required for a positive interpretation of EGFR and CK5/6. The NF-κB staining demonstrated a nuclear staining pattern, and if more than 10% of tumor cells stained, the specimen was considered to be positive.

### FISH for HER2 amplification

The HER2 DNA probe kit (PathVysion; Vysis, Downers Grove, IL, USA) included two DNA probes directly labeled with different fluorescent dyes: the Spectrum-Orange fluorophore-labeled HER2 (190 kb) specific for the HER2 gene locus on chromosome 17q11.2-q12, and the Spectrum-Green fluorophore-labeled chromosome enumerator probe (5.4 kb) targeting the alpha satellite DNA sequence located at the centromeric region of chromosome 17 (CEP17; 17p11.1-q11.1).

FISH assay was performed according to the protocol provided by Vysis (Downers Grove, IL, USA). Routinely processed paraffin-embedded tissue sections (4 μm) were deparaffinized in three changes of fresh xylene for three minutes each, dehydrated in two changes of 100% ethanol for three minutes each, and then allowed to air dry. Slides were then placed in a preheated 80°C pretreatment reagent (1 M sodium isothiocyanate;Vysis, Downers Grove, IL, USA) for 13 minutes, rinsed in distilled water for three minutes, and allowed to air dry. Protease digestion was accomplished by placing the slides in a prewarmed 37°C protease solution (Vysis, Downers Grove, IL, USA) for 13 minutes. Samples were then rinsed in distilled water for three minutes and air-dried. All slides were hybridized under identical conditions and with appropriate control tissue. An aliquot of pre-diluted LSI HER-2 Spectrum-Orange/CEP17 Spectrum-Green (Vysis, Downers Grove, IL, USA) was applied to the region of interest on the slide. A coverslip was placed and sealed at the periphery with rubber cement. The slides were placed on a preprogrammed, humidified slide warmer (Hybrite; Vysis, Downers Grove, IL, USA) with the following settings: denaturation at 73°C for five minutes and hybridization at 37°C for 16 hours. After hybridization, the rubber cement was removed, and the coverslip was floated off by soaking the slides in two times standard saline citrate buffer with 0.3% Nonidet P-40 (2 × SSC/0.3% NP-40) at ambient temperature. The slides were incubated in pre-warmed 2 × SSC/0.3% NP-40 at 73°C for two minutes, immersed in 2× SSC/0.3% NP-40 at ambient temperature for one minute, air dried in the dark, counterstained with 7 ml of 4,6-diamidino-2-phenylindole dihydrochloride (DAPI; Vysis, Downers Grove, IL, USA), and a coverslip was placed on the slide.

The signals were enumerated using an epifluorescence microscope (Olympus AX70; Melville, NY, USA) fitted with a Spectrum-Orange, Spectrum-Green, and DAPI triple-filter set. At least 60 cells were scored in each preparation, and the copy numbers of HER2 and CEP17 for each cell were recorded. HER2 was quantified using the ratio of HER2 to CEP17 signal counts. HER2 gene amplification was defined as an HER2 to CEP17 signal ratio of 2.0 or greater. Polysomy of chromosome 17 was defined as the presence of three or more CEP17 signals in more than 6% of the tumor cells evaluated.

### IHC subtyping criteria

The IHC-based definition of breast cancer subtypes used in this study was as follows: luminal A (ER+ and/or PR+, HER2-), luminal B (ER+ and/or PR+, HER2+), HER2+/ER- (ER-, PR-, HER2+), and basal-like (ER-, PR-, HER2-, CK5/6+) [9].

### Statistical analysis

Descriptive statistics were calculated. Fisher's exact tests were used to assess the association between categorical variables. P values (two-sided test) of less than 0.05 were considered significant. All statistical analyses were carried out using SAS 8.0 (SAS Institute, Cary, NC, USA).

## Results

The clinicopathological features of breast carcinoma from 42 men are summarized in Table 2. The racial distribution was as follows: 28 Caucasians, seven African Americans and seven Hispanics. Ages of the patients ranged from 33 to 82 years with a mean age of 63 years. Patients with luminal B subtype tumor was slightly younger than those with luminal A subtype (mean age 58 vs. 64 years, respectively), but the difference was not statistically significant (*P *= 0.25). Most of the patients were diagnosed with stage I to II disease (81%, 34/42) and were treated surgically (95%, 40/42), either by lumpectomy or mastectomy. Many patients also received other treatments including hormonal therapy (88%, 36/42), chemotherapy (64%, 27/42, with 2/27 received pre-operatively), and radiation therapy (19%, 8/42). There were no significant differences identified between the subtypes of male breast cancer regarding patients' race, presence of distant metastasis, and clinical stage.

**Table 2:** Clinicopathologic characteristics of subtypes

Subtypes	Luminal A (n = 35)	Luminal B (n = 7)	Total (n = 42)	*P *value*
Demographics				
Mean age (years)	64	58	63	*0.25*
Race				
Caucasian	22	6	28	
African American	6	1	7	
Hispanic	7	0	7	

Histologic type				
Invasive ductal carcinoma	30	7	37 (88%)	
Invasive papillary carcinoma	5	0	5 (12%)	
Other types	0	0	0	

Nuclear grade				*0.06*
1	2	0	2	
2	21	2	23	
3	12	5	17	

Cases with positive lymph node	14 (41%)	5 (71%)	19 (45%)	*0.40*

Mean number of positive lymph nodes	1.5	2.3	1.6	*0.40*

Stage grouping				
I	9	1	10	
IIA	10	3	13	
IIB	9	2	11	
IIIA	2	0	2	
IIIB	4	1	5	
IV	1	0	1	

* Fisher's exact test.

### Histologic features of subtypes

The histologic variants of male breast carcinoma were as follows: 37 (88%) were invasive ductal carcinomas and five were invasive papillary carcinomas (12%). All seven tumors of luminal B subtype were histologically invasive ductal carcinoma. Invasive papillary carcinomas were seen only in luminal A subtype (Table 2).

**Figure 1:**
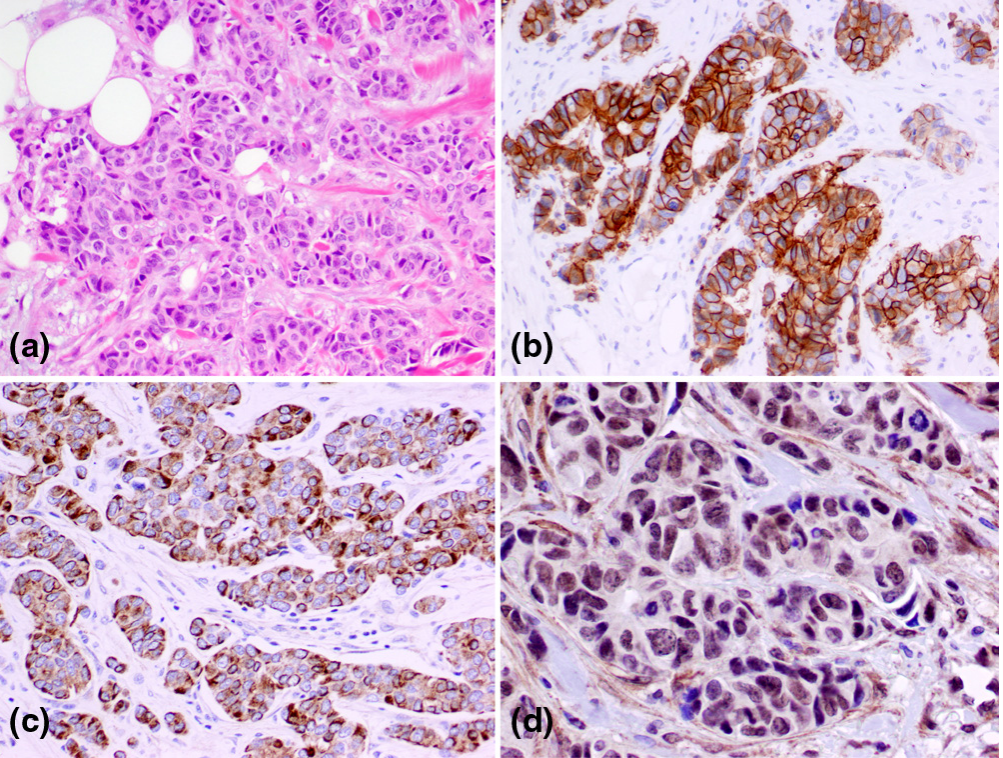
Photographs of a luminal B subtype of male breast cancer with H&E immunohistochemical stains. The luminal B tumor demonstrated histologic features of **(a) **invasive ductal carcinoma with high nuclear grade. The tumor showed **(b) **strong membraneous stain (3+) of human epidermal growth factor receptor 2 (HER2), **(c) **diffuse cytoplasmic staining of epidermal growth factor receptor (EGFR), and **(d) **nuclear staining of nuclear factor-κB (NF-κB).

More than half of the luminal A tumors (60%, 21/35) had intermediate nuclear grade (modified Black's nuclear grade 2) and one-third of them (34%, 12/35) had high nuclear grade (grade 3). Low nuclear grade tumors (nuclear grade 1) were seen only in luminal A subtype (n = 2). In comparison, high nuclear grade (grade 3) tumors were more frequent in luminal B subtype (71%, 5/7, Figure 1a) than in luminal A subtype (*P *= 0.09, Table 2).

Patients with luminal B subtype carcinomas had an increased tendency to have lymph node involvement (71%, 5/7) compared with patients with luminal A subtype tumors (41%, 14/35). Furthermore, the mean number of positive lymph nodes observed in luminal B subtype (2.2) was slightly higher than that seen in luminal A subtype (1.8). None of these differences were statistically significant (Table 2).

### Immunostain profile of male breast cancer

The expression frequencies of the immunomarkers in our male breast carcinoma samples are summarized in Table 3. ER was expressed in all tumors, while 64% of them were PR+. Among the tumors tested equivocal (++) or positive (+++) for HER2 on immunostain, seven were confirmed by positive FISH tests with HER2-to-CEP17 signal ratio of 2.0 or more. Based on the immunophenotyping and FISH study, the most common subtype in our group of male breast carcinoma was luminal A subtype (78%, 33/42), which was followed by luminal B subtype (17%, 7/42). There were no HER2+/ER-, basal-like, or unclassified subtypes observed in this group. Luminal A tumors tended to have a slight higher frequency of PR expression (69%, 24/35) than luminal B subtype (43%, 3/7). CK5/6 was expressed in 12% of the male breast carcinomas (Table 3), and expression was slightly more common in luminal B tumors (43%, 3/7) than luminal A subtype (26%, 9/35). By definition, over-expression and amplification of HER2 was demonstrated in all seven luminal B tumors (Figure 1b).

**Table 3:** Immunohistochemical results of male breast carcinoma

Immunostains	Luminal A n = 35	Luminal B n = 7	Total	*P *value*
ER	35	7	42 (100%)	
PR	24	3	27 (64%)	0.23
HER2	0	7	7 (16%)	
CK 5/6	9	3	12 (28%)	0.39
EGFR	6	3	9 (21%)	0.16
NF-κB	13	4	17 (40%)	0.41

* Fisher's exact test.

CK = cytokeratin; EGFR = epidermal growth factor receptor; ER = estrogen receptor; HER2 = human epidermal growth factor receptor 2; NF-κB = nuclear factor-κB; PR = progesterone receptor.

Positive EGFR stain was observed in 21% (9/42) of the samples; this staining was more frequently seen in carcinoma of luminal B subtype (42%, 3/7) than in luminal A subtype (17%, 6/35, Table 3). The staining pattern of EGFR in luminal B subtype tumors was diffuse and strong (Figure 1c), frequently intensifying at the leading edges of tumor invasion. In contrast, EGFR staining in luminal A subtype carcinoma was weak and focal. NF-κB nuclear staining was demonstrated in 40% (17/42) of tumors (Figure 1d). A higher frequency of NF-κB expression was observed in tumors of luminal B subtype (57%, 4/7) than those of luminal A subtype (37%, 13/35, Table 3). However, significant differences in expression of above immunomarkers were not observed between the two subtypes, probably owing to the small study group (Table 3).

## Discussion

Although the molecular subtypes of breast cancer were originally identified by gene expression analysis using DNA microarrays, IHC markers have been used as surrogates for DNA microarray in subtyping breast cancer. Based on recent updated IHC subtype definitions [9], we identified two molecular subtypes of male breast carcinoma in our group as luminal A (83%) and luminal B (17%) subtypes. Basal-like, HER2+/ER-, and unclassified subtypes were not observed in this group. This distribution differs from large study series in female breast carcinoma [8,9,24,25], which reported much lower frequencies of luminal A subtype (51 to 69%) but significantly higher frequencies of basal-like (12 to 21%) and HER2+/ER- (7 to 12%) subtypes. Our study also showed that male breast carcinomas in this group universally expressed ER (100%), a significantly higher frequency than that in female breast carcinomas (60 to 69%) [4,9]. This result was in agreement with previous studies showing that male breast carcinoma has a higher percentage of ER positivity (81 to 100%) than female breast cancer or gynecomastia [4,23,26,27]. The knowledge of immunophenotype-based subtyping of male breast carcinoma may be applied in comparison of treatment responses and prognosis in matched subtypes of female breast carcinoma.

The *HER2/neu *gene is a member of a gene family encoding transmembrane receptors for growth factors, including EGFR, HER2, HER3, and HER4. Approximately 25 to 30% of invasive female breast cancers over-express HER2 [28]. However, studies of HER2 over-expression in male breast cancer are limited with conflicting results. Pooled data demonstrated that HER2 over-expression detected by IHC was in a range of 2 to 56% with an average of 23% (reviewed in [29]). The inconsistency in percentage of positive HER2 immunostain may be due to differences in antibody preparation, scoring systems, and cut-off values used in these studies. A recent study of 99 cases of male breast cancer demonstrated that 15% of the tumors were 2+ (equivocal) or 3+ (positive) on HER2 immunostain and 11% were positive using FISH analysis [29]. In the current study, we detected 7 of 42 (16%) of male breast cancers with HER2 gene amplification determined using both IHC (3+) and FISH analysis. Although the early data tended to show higher percentages of positive HER2 immunostain, we believe that true HER2-positive cases in male breast cancer are probably fewer than those of female breast carcinoma when the current American Society of Clinical Oncology/College of American Pathologists guidelines for IHC with FISH analysis are applied.

Over-expression of HER2 is a well-known prognostic factor associated with poor survival in women with breast carcinoma [5,28]. However, the data are limited in male breast cancer regarding the association between HER2 over-expression and survival. In an early analysis of 17 cases of invasive male breast cancer, HER2 over-expression was detected by IHC in seven (41%) patients who were associated with significantly shortened survival [30]. Nevertheless, in a recent study, male breast cancers failed to show an association between HER2 over-expression and lymph node metastasis, a known unfavorable prognostic factor [29]. In our study, the luminal B subtype tumors (phenotype HER2+/ER+ and/or PR+) were more frequently associated with high nuclear grade, an unfavorable factor in both women and men [31]. More frequently, patients with luminal B subtype tumors had nodal metastasis and an increased number of positive lymph nodes. Although these findings suggest that carcinoma with HER2 over-expression may be associated with unfavorable prognostic factors, the differences were statistically insignificant due to our small cohort and a relatively short period of follow-up. Obviously, a high percentage of ER expression in male breast carcinoma is inevitably associated with the rarity of HER2+/ER- subtype carcinoma, which reportedly has a worse prognosis than that of luminal B tumors (HER2+/ER+ or PR+) in female patients. This may raise questions on the correlation in prognosis of the breast cancer subtypes between males and females. It is unknown whether the paucity or absence of the unfavorable breast carcinoma subtypes in males, such as HER2+/ER-, basal-like, or unclassified subtypes, is associated with a favorable prognosis. It is equally important whether the same subtypes of breast carcinoma have similar responses to therapy in males compared with females. To answer these questions, further investigation by comparison of prognosis and therapy responses with matched subtypes of breast carcinoma in males and females is warranted.

Based on studies in female patients with breast cancer, EGFR was reportedly over-expressed in aggressive [10-13] and metastatic breast carcinomas [32]. In breast carcinomas with EGFR over-expression, NF-κB was reportedly activated [17], which was also linked to unfavorable prognosis by promoting tumor metastasis and inhibiting apoptosis [18,20]. The data on EGFR expression in male breast cancer are very limited with only a few earlier studies conducted more than a decade ago. The reported expression rate of EGFR in male breast carcinomas varied significantly from 8.5% to 76% [31,33,34], probably due to variations in antibody preparation, staining protocol, and interpretation criteria. In our study, 21% of the tumors expressed EGFR, which is in agreement with one of the previous studies (20%) [31]. Interestingly, we found stronger EGFR staining was present at the leading edge of tumor invasion in high-grade tumors of luminal B subtype. We speculate that the higher expression rates for EGFR and NF-κB in luminal B subtype is an important factor contributing to their aggressive behavior and poor clinical prognosis. However, although higher expression rates of EGFR and NF-κB were observed in the luminal B subtype than those in the luminal A subtype, the difference lacked statistical significance due to the small study group.

In summary, the current study attempts to characterize subtypes of male breast cancer by using IHC markers. It provides new information for future study on prognosis, drug trials, and clinical management of breast cancer in men. However, our data need to be interpreted with caution for several reasons. Firstly, the study was designed on the basis of published data from female breast cancer that allowed subclassification of these tumors according to their characteristic features in DNA microarray and expression profiles [5,7]. DNA microarray data on male breast cancer are currently not available because of the rarity of this malignancy. Therefore, it is uncertain whether the gender differences present in these tumors regarding the genetic features and associated expression profiles. Secondly, the current study was unable to provide correlative data between the immunosubtypes of male breast cancer and their clinical behavior and survival information due to a relatively short period of follow-up. It is certainly critical in future studies to identify the subtypes of male breast carcinoma and their association with survival as well as the candidate markers with potential prognostic and predictive values. Finally, due to the rarity of the disease, the small number of cases collected in this group may affect validation of its conclusion and the statistical power of the observations. Additional studies with larger numbers of patients are needed to achieve sufficient statistical power.

## Conclusions

Our study demonstrates that luminal A and luminal B subtypes are the major subtypes of male breast carcinoma. The immunophenotypical features of male breast cancer differ from those of its female counterpart. Luminal B tumors tend to have high nuclear grade and more frequent expression of EGFR and NF-κB.

## Abbreviations

CEP: centromeric region of chromosome 17; CK: cytokeratin; DAPI: 4,6-diamidino-2-phenylindole dihydrochloride; EGFR: epidermal growth factor receptor; ER: estrogen receptor; FISH: fluorescent in situ hybridization; HER2: human epidermal growth factor receptor 2; IHC: immunohistochemistry; NF: nuclear factor; PR: progesterone receptor.

## Competing interests

The authors declare that they have no competing interests.

## Authors' contributions

YMG and MG were responsible for study design, data analyses, manuscript preparation, and editing. YG, NS, MAE, and ZW made substantial contributions to the study and to the interpretation of data. EL was responsible for statistical analyses of data. All authors read and approved the final manuscript.
